# The Chromatin Remodeling Protein BRG1 Regulates SREBP Maturation by Activating SCAP Transcription in Hepatocytes

**DOI:** 10.3389/fcell.2021.622866

**Published:** 2021-02-25

**Authors:** Ming Kong, Yuwen Zhu, Jing Shao, Zhiwen Fan, Yong Xu

**Affiliations:** ^1^Key Laboratory of Targeted Intervention of Cardiovascular Disease and Collaborative Innovation Center for Cardiovascular Translational Medicine, Department of Pathophysiology, School of Basic Medical Sciences, Nanjing Medical University, Nanjing, China; ^2^Wu Medical School, Jiangnan University, Wuxi, China; ^3^Department of Pathology, Affiliated Nanjing Drum Tower Hospital of Nanjing University School of Medicine, Nanjing, China; ^4^Institute of Biomedical Research, Liaocheng University, Liaocheng, China

**Keywords:** transcriptional regulation, hepatocyte, lipid metabolism, transcription factor, chromatin remodeling protein, steatosis

## Abstract

Sterol response element binding protein (SREBP) is a master regulator of cellular lipogenesis. One key step in the regulation of SREBP activity is its sequential cleavage and *trans-*location by several different proteinases including SREBP cleavage activating protein (SCAP). We have previously reported that Brahma related gene 1 (BRG1) directly interacts with SREBP1c and SREBP2 to activate pro-lipogenic transcription in hepatocytes. We report here that BRG1 deficiency resulted in reduced processing and nuclear accumulation of SREBP in the murine livers in two different models of non-alcoholic steatohepatitis (NASH). Exposure of hepatocytes to lipopolysaccharide (LPS) and palmitate (PA) promoted SREBP accumulation in the nucleus whereas BRG1 knockdown or inhibition blocked SREBP maturation. Further analysis revealed that BRG1 played an essential role in the regulation of SCAP expression. Mechanistically, BRG1 interacted with Sp1 and directly bound to the SCAP promoter to activate SCAP transcription. Forced expression of exogenous SCAP partially rescued the deficiency in the expression of SREBP target genes in BRG1-null hepatocytes. In conclusion, our data uncover a novel mechanism by which BRG1 contributes to SREBP-dependent lipid metabolism.

## Introduction

Lipid is a major biological macromolecule that plays diverse roles in orchestrating differentiation, proliferation, migration, communication, survival, and death of mammalian cells (Glatz, 2011; Patil et al., 2019; Nechipurenko, 2020; Xie et al., 2020). On the other hand, disorders of lipid metabolism, typically characterized excessive lipid synthesis and defective lipid catabolism, contribute to a host of human diseases including coronary heart disease, obesity, hypertension, and non-alcoholic steatohepatitis/NASH (Cifkova, 2014; Islam et al., 2019; Magni, 2019; Pei et al., 2020). Liver is a major hub of lipid metabolism (Nguyen et al., 2008). The hepatocytes rely on thousands of proteins, which include transmembrane receptors, transcriptional regulators, and transporters, to coordinate lipid metabolism tailoring to various cellular events. This normally well-programmed process can be hijacked by both intrinsic and extrinsic pathogenic stimuli to skew lipid metabolism and to promote the development of diseases (Neuschwander-Tetri, 2010; Mashek et al., 2015).

Sterol response element binding protein (SREBP), initially identified and characterized by the Brown and Goldstein laboratory, represents a family of transcription factors considered master regulators of lipid metabolism (Shimano and Sato, 2017). Three members have been identified for this family, SREBP1a, SREBP1c, and SREBP2. It is generally agreed that SREBP1a/1c are primarily responsible for the synthesis of fatty acids whereas SREBP2 selectively regulates cholesterol synthesis although functional redundancies among the three SREBP isoforms have been observed (Horton et al., 2002b). All three SREBP proteins can be detected to exhibit a unanimous expression pattern but SREBP1c and SREBP2 are the predominantly expressed isoforms in most tissues *in vivo* (Shimomura et al., 1997). The relevance of SREBPs in lipid homeostasis and in the pathogenesis of lipid disorder-associated human diseases has been mostly supported by transgenic animal models. Deletion of SREBP1c in mice, for instance, results in approximately 50% reduction in hepatic fatty acid production accompanying an across-the-board down-regulation of enzymes involved in lipogenesis (Liang et al., 2002). On the contrary, mice harboring over-expression of either SREBP1c or SREBP2 develop fatty liver (steatosis) spontaneously although SREBP1c over-expression preferentially leads to an increase in plasma triglyceride levels whereas SREBP2 over-expression causes hypercholesterolemia (Horton et al., 2002a).

Innate SREBP proteins, once translated, dwell in the ER as *trans-*membrane factors (Goldstein et al., 2006). Processing and consequently nuclear translocation of SREBP entails the participation of three accessory proteins, SREBP cleavage activating protein (SCAP), site 1 protease (S1P), and site 2 protease (S2P). Upon stimulation by various factors, SCAP escorts SREBP to the Golgi apparatus where they are clipped, sequentially, by S1P and S2P (Brown and Goldstein, 1997). The liberated/mature SREBP, designated as nSREBP, moves into the nucleus functioning as a pro-lipogenic transcription factor to regulate lipid homeostasis and disorder (Hampton, 2002). Consistent with this model, liver specific deletion of SCAP results in defective processing of all three nSREBPs and a drastic reduction in hepatic lipid production (Horton et al., 2002a). In addition, genetic polymorphism of the *SCAP* gene has been found to be associated with NASH and hypertension in humans (Sun et al., 2013; Yang et al., 2017). However, the epigenetic regulation of *SCAP* transcription during NASH pathogenesis remains poorly defined.

Brahma related gene 1 (BRG1) utilizes the energy derived from ATP hydrolysis to mobilize nucleosomes along the chromatin and modulate chromatin accessibility. We have previously shown that BRG1 is an important moderator of liver injury linking specific transcriptional events to the alteration of liver function (Fan et al., 2019; Li et al., 2019a, c, d, e; Dong et al., 2020; Shao et al., 2020). Importantly, liver specific deletion of BRG1 attenuates steatosis in several different animal models (Kong et al., 2018; Li et al., 2018; Liu et al., 2019a; Fan et al., 2020). Here we describe a novel mechanism whereby BRG1 contributes to hepatic liver metabolism by regulating SCAP-mediated SREBP processing.

## Materials and Methods

### Animals

All animal experiments were reviewed and approved by the intramural Ethics Committee on Humane Treatment of Experimental Animals. *Smarca4*^*f/f*^ mice (Dong et al., 2020) were crossed to *Alb*-Cre mice (Kong et al., 2019b) to generate hepatocyte conditional BRG1 knockout (LKO) mice. To induce NASH, 8 week-old male mice were fed a high fat diet (D12492, Research Diets) for 16 consecutive weeks (Fan et al., 2019). Alternatively, 8 week-old male mice were fed a methionine- and choline-deficient (MCD) diet (A02082002B, Research Diets) for four consecutive weeks as previously described (Kong et al., 2019a).

### Cell Culture, Plasmids, Transient Transfection, and Reporter Assay

Primary mouse hepatocytes were isolated and maintained as previously described (Li et al., 2019a). HepG2 cells were maintained in DMEM supplemented with 10% FBS. The human SCAP promoter-luciferase constructs (Nakajima et al., 1999), FLAG-tagged BRG1, Myc-tagged SCAP (Chen et al., 2016), have been described previously. Small interfering RNAs targeting BRG1 are: #1, AACATGCACCAGATGCACAAG and #2, GCCCATGGAGTCCATGCAT. Transient transfection was performed with Lipofectamine 2000. Cells were harvested 48 h after transfection. Luciferase activities were assayed 24–48 h after transfection using a luciferase reporter assay system (Promega) as previously described (Yang et al., 2019a, b).

### Protein Extraction and Western Blot

Whole cell lysates were obtained by re-suspending cell pellets in RIPA buffer (50 mM Tris pH7.4, 150 mM NaCl, and 1% Triton X-100) with freshly added protease inhibitor (Roche) as previously described (Lv et al., 2020; Mao et al., 2020; Yang et al., 2020a, b). Nuclear proteins were prepared with the NE-PER Kit (Pierce) following manufacturer’s recommendation. Western blot analyses were performed with anti-BRG1 (Santa Cruz, sc-10768), anti-SREBP1 (Proteintech, 14088-1), anti-SREBP2 (Proteintech, 19811-1), anti-SCAP (Cell Signaling Tech, 13102), and anti-β-actin (Sigma, A2228) antibodies. For densitometrical quantification, densities of target proteins were normalized to those of b-actin as previously described (Sun et al., 2020; Wu T. et al., 2020). Data are expressed as relative protein levels compared to the control group which is arbitrarily set as one.

### RNA Isolation and Real-Time PCR

RNA was extracted with the RNeasy RNA isolation kit (Qiagen). Reverse transcriptase reactions were performed using a SuperScript First-strand Synthesis System (Invitrogen) as previously described (Liu et al., 2019b; Zhao et al., 2019). Real-time PCR reactions were performed on an ABI Prism 7500 system with the following primers: human *SCAP*, 5′-TCACGTTGCAGCCGTCTTCCTT-3′ and 5′-CAGGATGCCA ATCCAGACAACG-3′; human *BRG1*, 5′-TCATGTTGGCG AGCTATTTCC-3′ and 5′-GGTTCCGAAGTCTCAACGATG-3′; human *FASN*, 5′-CTTCCGAGATTCCATCCTACGC-3′ and 5′-TGGCAGTCAGGCTCACAAACG-3′; human *LDLR*, 5′-GAC GTGGCGTGAACATCTG-3′ and 5′-CTGGCAGGCAATGCTT TGG-3′; mouse *Scap*, 5′-CCGAGGATGACCCTGACTGA-3′ and 5′-AGAGCAGCCCATGGTTGTAGA-3′; mouse *S1p*, 5′-CT ACTATGGAGGAATGCCGACAG-3′ and 5′-CTCCGTTCTGT GGCAAATAGGG-3′; mouse *S2p*, 5′-ACGGCGGAAAGCAA GGATGCTT-3′ and 5′-GTGCCAAAGTCTGCATCAGCGT-3′; mouse *Fasn*, 5′-TTTAAAGGGAGGGAGGGAGA-3′ and 5′-GG CAGGATAGGGAAACACTGA-3′; mouse *Ldlr*, 5′-TGTGAAT TTGGTGGCTGAAAAC-3′ and 5′-AATAGGGAAGAAGATG GACAGGAAC-3′. Ct values of target genes were normalized to the Ct values of housekeekping control gene (18s, 5′-CGCGGTTCTATTTTGTTGGT-3′ and 5′-TCGTCTTCGAAACTCCGACT-3′ for both human and mouse genes) using the ΔΔCt method and expressed as relative mRNA expression levels compared to the control group which is arbitrarily set as one.

### Chromatin Immunoprecipitation

Chromatin immunoprecipitation (ChIP) assays were performed essentially as described before (Li et al., 2019b, f, 2020a, b, c; Shao et al., 2019; Weng et al., 2019; Chen et al., 2020a; Wu X. et al., 2020; Hong et al., 2021). In brief, chromatin in control and treated cells were cross-linked with 1% formaldehyde. Cells were incubated in lysis buffer (150 mM NaCl, 25 mM Tris pH 7.5, 1% Triton X-100, 0.1% SDS, and 0.5% deoxycholate) supplemented with protease inhibitor tablet and PMSF. DNA was fragmented into ∼200 bp pieces using a Branson 250 sonicator. Aliquots of lysates containing 200 μg of protein were used for each immunoprecipitation reaction with anti-BRG1 (Santa Cruz, sc-10768), anti-Sp1 (Abcam, ab227383), or pre-immune IgG. Precipitated genomic DNA was amplified by real-time PCR with the following primers: *SCAP* proximal promoter, 5′-ATACTTCCCTCCGGTGTCCAC-3′ and 5′-ACCTCTCACCTCCACCTTTAC-3′; *SCAP* distal promoter, 5′-AAATGCGAGGACATGTACAATAC-3′ and 5′-ATTTAAAAGCTAAGTTGAC-3′. A total of 10% of the starting material is also included as the input. Data are then normalized to the input and expressed as % recovery relative the input as previously described (Chen et al., 2020b, c). All experiments were performed in triplicate wells and repeated three times.

### Statistical Analysis

Sample sizes reflected the minimal number needed for statistical significance based on power analysis and prior experience. Two-tailed Student’s *t*-test was performed using an SPSS package. Unless otherwise specified, *p* values smaller than 0.05 were considered statistically significant.

## Results

### BRG1 Regulates SREBP Maturation in the Liver

**FIGURE 1:**
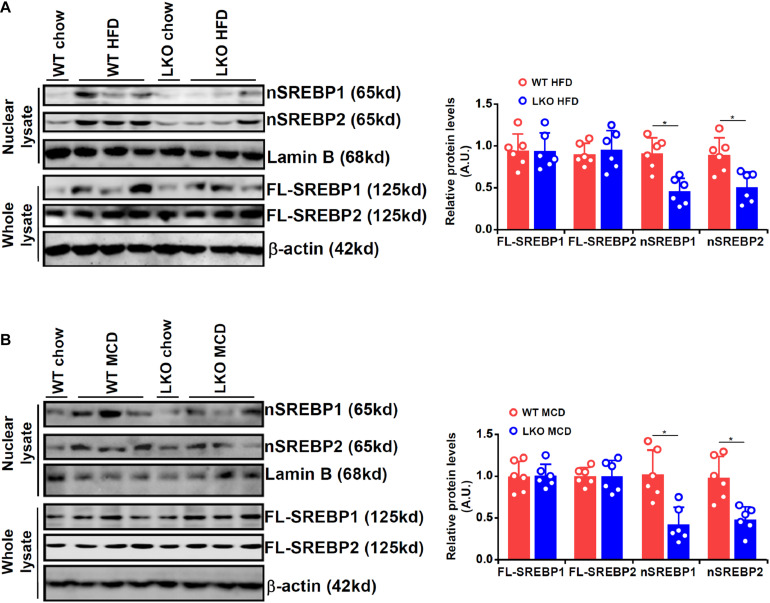
BRG1 regulates SREBP maturation in the liver. **(A)** WT and BRG1 LKO mice were fed a high-fat diet (HFD) for 16 weeks. SREBP levels were examined in whole liver lysates and liver nuclear lysates by Western blotting. **(B)** WT and BRG1 LKO mice were fed a methionine-and-choline deficient diet (MCD) for 4 weeks. SREBP levels were examined in whole liver lysates and liver nuclear lysates by Western blotting. *N* = 6 mice for each group. Data represent averages of three independent experiments and error bars represent SEM. *p < 0.05, two-tailed Student’s *t*-test.

We first evaluated the effect of BRG1 deficiency on SREBP maturation in the liver in two different models of steatosis. To this end, we generated liver conditional BRG1 knockout (LKO) mice by crossing the *Smarca4*-flox mice with the *Alb*-Cre mice. In the first model in which the mice were fed a high-fat diet (HFD) for 16 weeks, it was observed that nuclear SREBP1/2 levels were appreciably increased in the livers of the HFD-fed mice compared to the control diet-fed (chow) mice; up-regulation of nuclear SREBP1/2 by HFD feeding was more modest in the LKO livers than in the WT livers (Figure 1A). In the second model in which the mice were fed a methionine-and-choline deficient (MCD) diet for 4 weeks, a similar observation was made that BRG1 deficiency dampened the accumulation of SREBP1/2 in the nucleus.

### BRG1 Is Essential for SREBP Maturation in Hepatocytes

**FIGURE 2:**
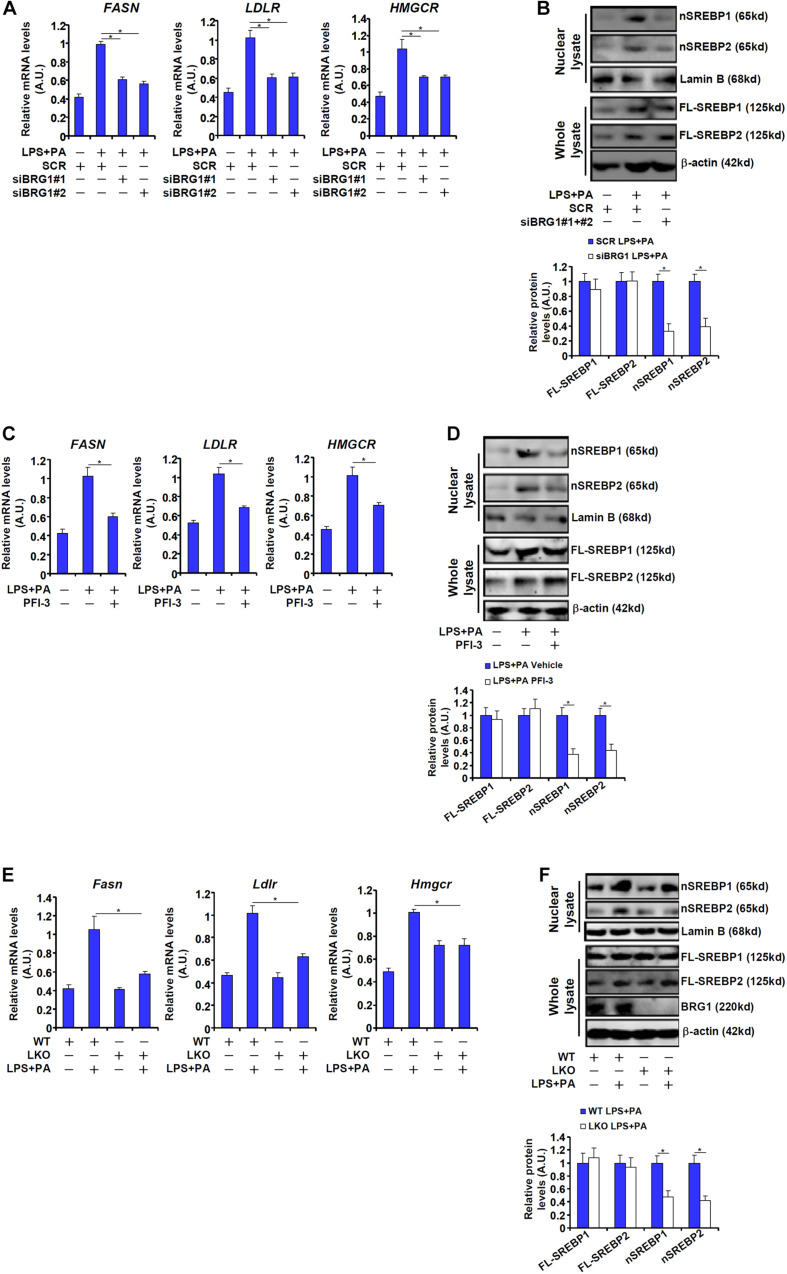
BRG1 is essential for SREBP maturation in hepatocytes. **(A,B)** HepG2 cells were transfected with siRNA targeting BRG1 or scrambled siRNA (SCR) followed by treatment with LPS (1 μg/ml) + PA (0.2 mM). SREBP target genes were examined by qPCR. SREBP levels were examined in whole cell lysates and nuclear lysates by Western blotting. **(C,D)** HepG2 cells were treated with LPS (1 μg/ml) + PA (0.2 mM) in the presence or absence of PFI-3 (3 μM). SREBP target genes were examined by qPCR. SREBP levels were examined in whole cell lysates and nuclear lysates by Western blotting. **(E,F)** Primary hepatocytes isolated from WT and BRG1 LKO mice were treated with LPS (1 μg/ml) + PA (0.2 mM). SREBP levels were examined in whole cell lysates and nuclear lysates by Western blotting. Data represent averages of three independent experiments and error bars represent SEM. *p < 0.05, two-tailed Student’s *t*-test.

We next verified the observation that BRG1 might contribute to SREBP maturation in cultured hepatocytes. The combined treatment of LPS plus free fatty acids (palmitate, PA), which has been reported to induce SREBP target gene expression in macrophages (Li et al., 2013), induced the expression of *FASN*, a prototypical SREBP1 target gene, and the expression of *LDLR*, prototypical SREBP2 target gene, in HepG2 cells (Figure 2A). Knockdown of BRG1 by siRNA attenuated the induction of both *FASN* and *LDLR*, indicative of repressed SREBP activity (Figure 2A). Consistent with these changes, we found that BRG1 knockdown reduced the levels of nSREBP1/2 without significantly altering full-length SREBP1/2 in LPS + PA-treated HepG2 cells (Figure 2B). Similarly, treatment with the small-molecule BRG1 inhibitor PFI-3 resulted in a down-regulation of SREBP target genes and partially blocked SREBP processing in HepG2 cells (Figures 2C,D). Finally, when primary hepatocytes were isolated from wild type and BRG1 LKO mice and exposed to LPS + PA treatment, the induction of SREBP target genes and SREBP maturation were attenuated in the LKO cells compared to the WT cells (Figures 2E,F).

### BRG1 Regulates SCAP Expression

**FIGURE 3:**
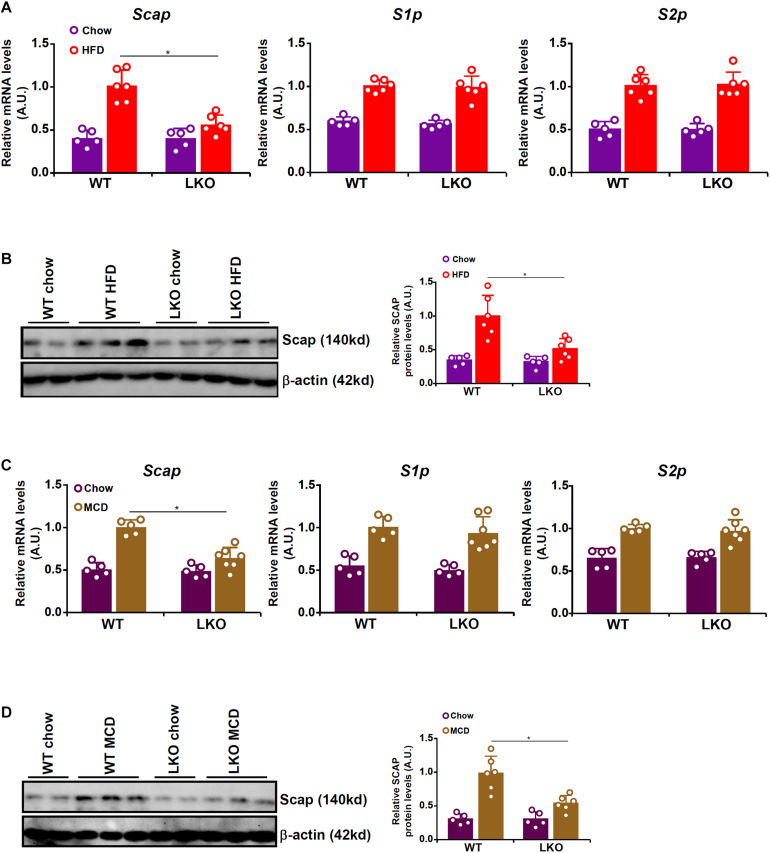
BRG1 regulates SCAP expression *in vivo*. **(A,B)** WT and BRG1 LKO mice were fed a high-fat diet (HFD) for 16 weeks. SCAP expression was examined by qPCR and Western blotting. *N* = 5 mice for the chow groups and *N* = 6 mice for the HFD groups. **(C,D)** WT and BRG1 LKO mice were fed a methionine-and-choline deficient diet (MCD) for 8 weeks. SCAP expression was examined by qPCR and Western blotting. *N* = 5 mice for the chow groups and *N* = 6 mice for the MCD groups. Data represent averages of three independent experiments and error bars represent SEM. *p < 0.05, two-tailed Student’s *t*-test.

Because BRG1 deficiency was associated with impaired SREBP maturation in hepatocytes, we hypothesized that BRG1 might play a role regulating the expression levels of SREBP processing enzyme(s). We therefore compared the expression of the three SREBP processing enzymes, SCAP, S1P, and S2P, in WT and LKO livers. As shown in Figures 3A,B, all three SREBP processing proteins were up-regulated in HFD-fed livers compared to the chow-fed livers; BRG1 deficiency, however, dampened the up-regulation of SCAP expression without altering either S1P or S2P expression. Similarly, MCD diet feeding induced the mRNA (Figure 3C) and protein (Figure 3D) levels of SCAP, S1P, and S2P in the murine livers; the induction of SCAP expression by MCD diet feeding was more modest in the LKO livers than the WT livers whereas the induction of S1P/S2P expression was comparable between the LKO livers and WT livers.

**FIGURE 4:**
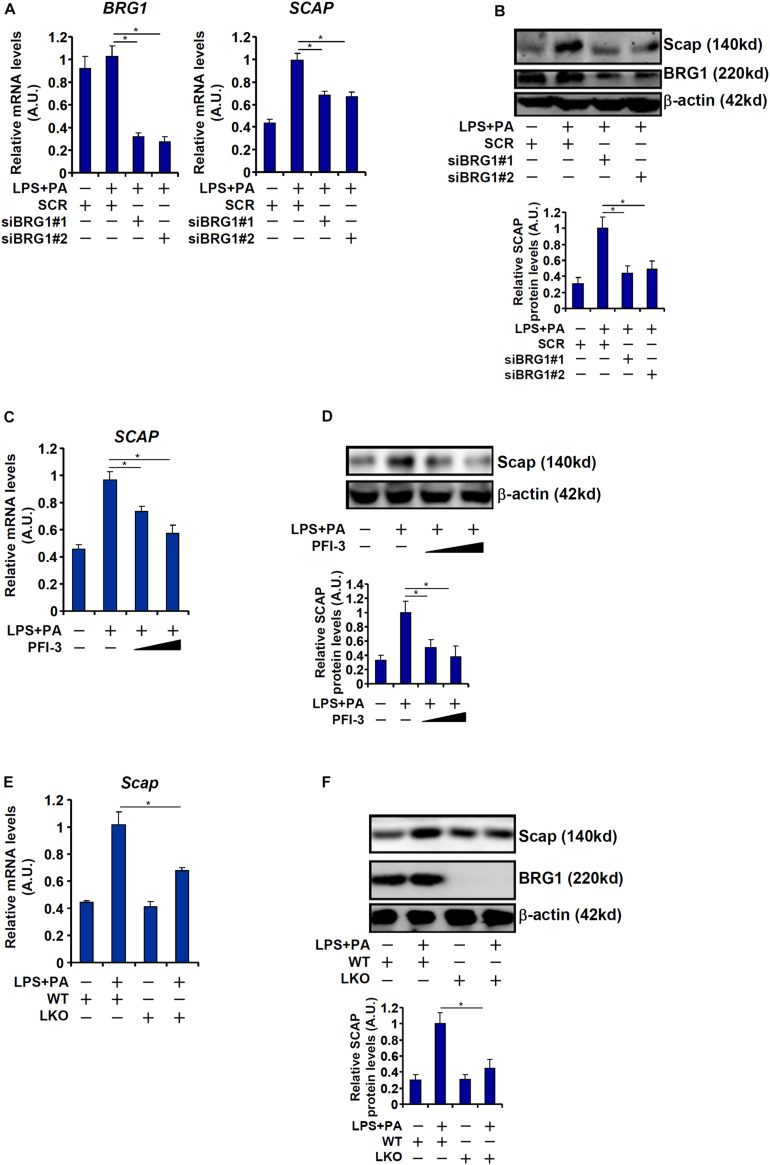
BRG1 regulates SCAP expression *in vitro*. **(A,B)** HepG2 cells were transfected with siRNA targeting BRG1 or scrambled siRNA (SCR) followed by treatment with LPS (1 μg/ml) + PA (0.2 mM). SREBP target genes were examined by qPCR. SCAP expression was examined by qPCR and Western blotting. **(C,D**) HepG2 cells were treated with LPS (1 μg/ml) + PA (0.2 mM) in the presence or absence of PFI-3 (3 μM). SCAP expression was examined by qPCR and Western blotting. **(E,F)** Primary hepatocytes isolated from WT and BRG1 LKO mice were treated with LPS (1 μg/ml) + PA (0.2 mM). SCAP expression was examined by qPCR and Western blotting. Data represent averages of three independent experiments and error bars represent SEM. *p < 0.05, two-tailed Student’s *t*-test.

Next, we evaluated the effect of BRG1 depletion or inhibition on SCAP expression in cultured hepatocytes. Exposure of HepG2 cells to LPS + PA treatment robustly augmented SCAP expression at both mRNA (Figure 4A) and protein (Figure 4B) levels. BRG1 knockdown by two independent siRNAs attenuated the induction of SCAP expression by LPS + PA treatment. Alternatively, the addition of a small-molecule BRG1 inhibitor (PFI-3) dose-dependently ameliorated the induction of SCAP expression by LPS + PA treatment (Figures 4C,D). Finally, when primary hepatocytes were isolated from the WT and the LKO mice and exposed to LPS + PA stimulation, the induction of SCAP molecules was not as strong in the LKO cells as in the WT cells (Figures 4E,F). Consistent with the changes in SCAP expression, it was also observed that there were fewer lipid droplets in the LKO hepatocytes than in the WT hepatocytes exposed to LPS + PA treatment (Supplementary Figure 1).

### BRG1 Interacts With Sp1 to Activate SCAP Transcription in Hepatocytes

**FIGURE 5:**
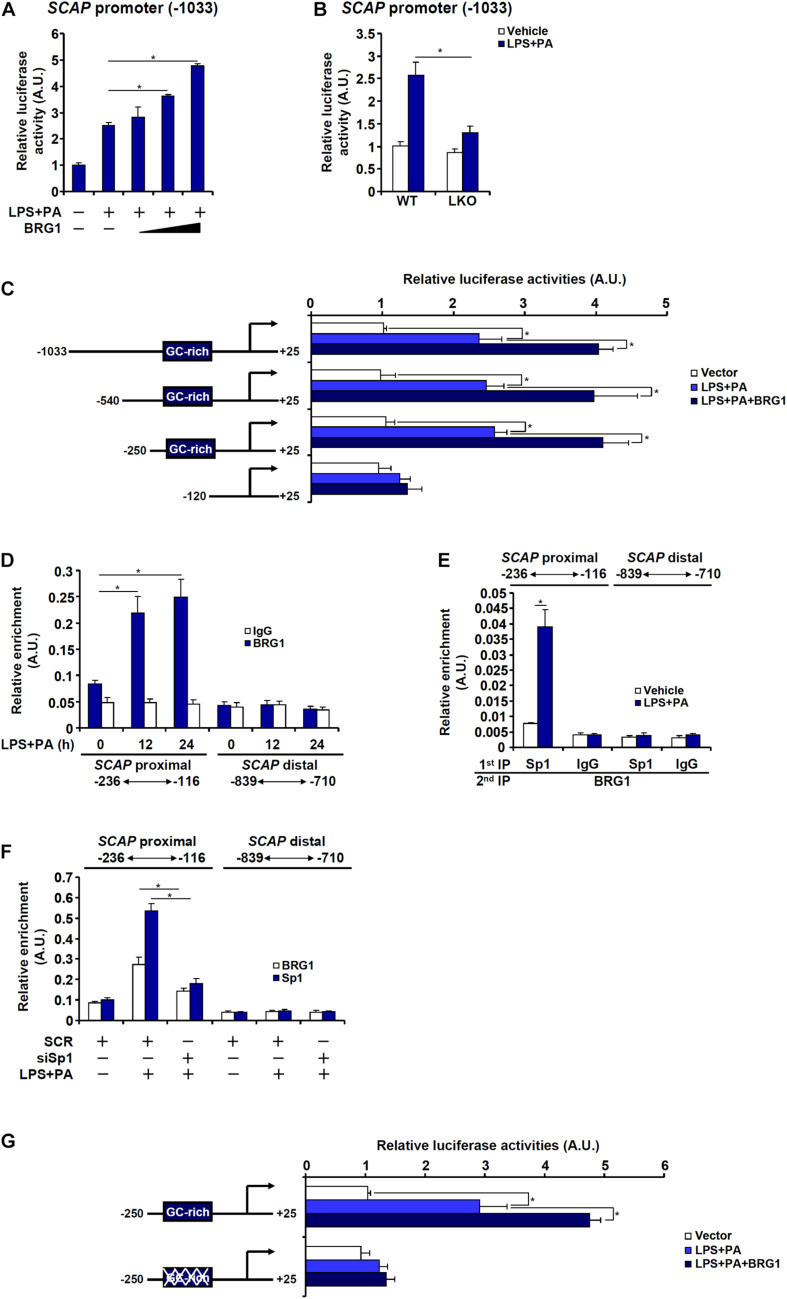
BRG1 interacts with Sp1 to activate SCAP transcription in hepatocytes. **(A)** An SCAP promoter-luciferase construct (–1033/+25) was transfected into HepG2 cells with or without BRG1 followed by treatment with LPS (1 μg/ml) + PA (0.2 mM). Luciferase activities were normalized by protein concentration and GFP fluorescence. **(B)** An SCAP promoter-luciferase construct (–1033/+25) was transfected into primary hepatocytes isolated from WT and BRG1 LKO mice followed by treatment with LPS (1 μg/ml) + PA (0.2 mM). Luciferase activities were normalized by protein concentration and GFP fluorescence. **(C)** SCAP promoter-luciferase constructs of various lengths were transfected into HepG2 cells with or without BRG1 followed by treatment with LPS (1 μg/ml) + PA (0.2 mM). Luciferase activities were normalized by protein concentration and GFP fluorescence. **(D)** HepG2 cells were treated with or without LPS (1 μg/ml) + PA (0.2 mM) and harvested at indicated time points. ChIP assays were performed with anti-BRG1 or IgG. **(E)** HepG2 cells were treated with or without LPS (1 μg/ml) + PA (0.2 mM) for 24 h. Re-ChIP assays were performed with indicated antibodies. **(F)** HepG2 cells were transfected with siRNA targeting Sp1 or scrambled siRNA (SCR) followed by treatment with LPS (1 μg/ml) + PA (0.2 mM). ChIP assays were performed with anti-Sp1 or anti-BRG1. **(G)** Wild type or Sp1 site mutant SCAP promoter-luciferase construct was transfected into HepG2 cells with or without BRG1 followed by treatment with LPS (1 μg/ml) + PA (0.2 mM). Luciferase activities were normalized by protein concentration and GFP fluorescence. Data represent averages of three independent experiments and error bars represent SEM. *p < 0.05, two-tailed Student’s *t*-test.

Next, it was determined whether regulation of SCAP expression by BRG1 in hepatocytes occurred at the transcriptional level. An SCAP promoter-luciferase fusion construct (−1,033) was transfected into HepG2 cells. Treatment with LPS + PA stimulated the SCAP promoter activity by more than twofold and BRG1 over-expression further augmented the SCAP promoter activity in a dose-dependent manner (Figure 5A). When the same SCAP promoter-luciferase construct was transfected into primary hepatocytes isolated from WT and BRG1 LKO mice, treatment with LPS + PA stimulated the SCAP promoter activity much more potently in the WT hepatocytes than in the LKO hepatocytes (Figure 5B). Serial deletions were then introduced to the full-length SCAP promoter-luciferase construct and the mutated constructs were tested for the responsiveness to the stimulation of LPS + PA treatment plus BRG1 over-expression. As shown in Figure 5C, the −540 construct and the −250 construct, but not the −120 construct, responded to the stimulation of LPS + PA treatment plus BRG1 over-expression comparably as the −1,033 construct. ChIP assay confirmed that LPS + PA treatment induced BRG1 recruitment to the SCAP proximal promoter between −250 and −120; as a negative control, BRG1 occupancy on the SCAP distal promoter was not detected with or without LPS + PA treatment (Figure 5D). Further analysis revealed a GC-rich region that could potentially occupied by the transcription factor Sp1 between −250 and −120 of the SCAP promoter. Furthermore, Re-ChIP assay showed that an Sp1-BRG1 complex was detectable on the SCAP promoter only when the cells were stimulated by LPS + PA (Figure 5E). ChIP assay confirmed that Sp1 knockdown abrogated the binding of both Sp1 and BRG1 to the proximal SCAP promoter (Figure 5F). Adding further support to the model that Sp1 recruits BRG1 to activate SCAP transcription was the observation that the SCAP promoter construct with Sp1 site mutated could not be induced by BRG1 over-expression (Figure 5G).

### SCAP Re-introduction Partially Rescues the Expression of SREBP Target Genes in BRG1 Deficient Hepatocytes

**FIGURE 6:**
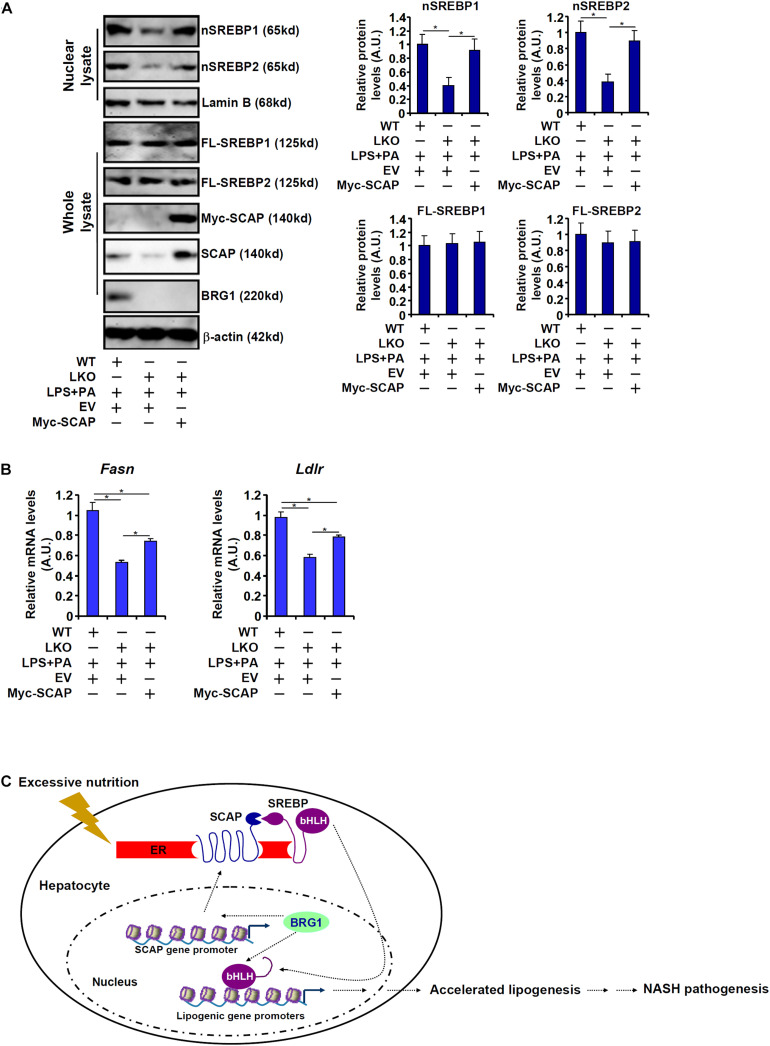
SCAP re-introduction partially rescues the expression of SREBP target genes in BRG1 deficient hepatocytes. **(A,B)** A Myc-tagged SCAP expression construct was transfected into primary hepatocytes isolated from BRG1 LKO mice followed by treatment with LPS (1 μg/ml) + PA (0.2 mM). Gene expression levels were examined by qPCR and Western blotting. Data represent averages of three independent experiments and error bars represent SEM. *p < 0.05, two-tailed Student’s *t*-test. **(C)** A schematic model. BRG1 may contribute to pro-lipogenic transcription in hepatocytes *via* at least two independent mechanisms. On the one hand, BRG1 stimulates SCAP expression to promote SREBP maturation. On the other hand, BRG1 interacts with mature SREBPs in the nucleus to directly activate pro-lipogenic gene transcription. Consequently, accelerated lipogenesis in hepatocytes leads to steatosis.

Finally, exogenous SCAP was introduced into BRG1-deficient hepatocytes to address the question as to whether it could rescue the expression of SREBP target genes. As shown in Figure 6A, over-expression of Myc-tagged SCAP largely restored SREBP processing in the LKO hepatocytes despite the absence of BRG1. Consistent with this observation, SCAP over-expression also partially normalized the levels of SREBP target genes: there was a 39% increase in *Fasn* expression and a 36% increase in *Ldlr* expression in the SCAP over-expressed LKO cells compared to the mock over-expressed LKO cells (Figure 6B). Accordingly, forced expression of exogenous SCAP partially restored accumulation of lipid droplet in the LKO hepatocytes (Supplementary Figure 2).

## Discussion

The *trans-*location and subsequent cleavage of nascent SREBPs catapults these master regulators of lipid metabolism into the nucleus to orchestrate pro-lipogenic transcription. This process is mediated by the well-conserved SCAP-S1P-S2P axis. We have previously shown that the chromatin BRG1 contributes to hepatic lipid metabolism by functioning as a co-activator for SREBP1/2 (Li et al., 2018; Fan et al., 2020). Here we detail a novel mechanism in which BRG1 modulates SREBP maturation *via* activating SCAP transcription (Figure 6C). Our data add to a growing body of evidence that cements the crucial role BRG1 plays in regulating cellular metabolism. For instance, Miao et al. (2009) have reported that BRG1 is essential for the synthesis of bile acids, a key function of hepatocyte, by interacting with the nuclear receptor FXR. Imbalzano and colleagues have demonstrated that BRG1 activates the transcription of several enzymes involved in fatty acid synthesis, including ATP citrate lyase (ACLY) and acetyl CoA carboxylase (ACC), to drive breast cancer proliferation (Wu et al., 2016; Nickerson et al., 2017). Metabolomic analysis has revealed that BRG1 deficiency in cardiomyocytes results in skewed fatty acid utilization, glycolysis, and glycogen synthesis (Banerjee et al., 2015). Genomewide transcriptomic studies suggest that BRG1 preferentially, at least in epithelial cells, binds to the SREBP target promoters to remodel chromatin structure (Barutcu et al., 2016). It is interesting to note that BRG1 deficiency or inhibition did not alter basal levels of SCAP in the livers (Figure 3) and in cultured hepatocytes (Figure 4), suggesting that BRG1 is dispensable for the maintenance of lipid homeostasis under physiological conditions. This observation is consistent with our previous findings showing that basal lipid profiles (triglycerides and cholesterol) are comparable between the WT mice and the BRG1 LKO mice. Rather, stimuli-induced SCAP expression appeared to rely on BRG1 suggesting that BRG1 is a pathogenic factor-driven regulator of lipid metabolism disorders. These data present Brg1 as an attractive target for intervention: since the absence of Brg1 presumably will not interfere with normal tissue/organ function, the predicament of selective drug delivery could be effectively circumvented.

Another noteworthy finding is that although SCAP re-introduction in the BRG1 LKO cells largely restored SREBP processing (Figure 6A), SREBP target gene expression was only partially, but not completely, corrected (Figure 6B). These data allude to a model in which SCAP-mediated SREBP maturation and nuclear translocation serves as a necessary but not adequate step for pro-lipogenic transcription. Indeed, several epigenetic factors have been shown to interact with BRG1 to regulate SREBP1 activity. The histone H4K16 acetyltransferase MOF (Liu et al., 2019a) and the H3K9 demethylase KDM3A can both bind to SREBP target promoters to regulate SREBP-dependent transcription (Fan et al., 2020). An intriguing question that remains to be answered is whether the functional relevance of MOF and/or KDM3A can be extended to the regulation of SCAP transcription and thus SREBP processing. There are a few indications wherein the *SCAP* promoter appears to be influenced by epigenetic modifications. Carraway et al. (2020) have shown that histone H3K9 acetylation and DNA methylation can differentially regulate SCAP expression in leukemia cells although the involvement of specific histone/DNA modifying enzymes is not clear at this point. It would be of great interest to further examine the epigenetic mechanism whereby SCAP transcription is regulated to provide more flexibility in targeting the SREBP pathway.

There are several lingering issues that deserve further attention in future studies. First, the broader biological/pathobiological significance of our finding, i.e., whether BRG1 can contribute to SREBP maturation in other pathophysiological conditions such as insulin stimulation or fast-feed cycle remains unclear. Of note, it has been previously shown that insulin-induced transcription of SREBP target genes in pre-adipocyte correlates with increased recruitment of BRG1 to the SREBP target promoters although it was not determined whether BRG1 could directly promote insulin-induced SREBP nuclear translocation (Lee et al., 2007). Second, we focused our study on the liver (hepatocytes), one of the three major peripheral tissues targeted by insulin. Because SREBP maturation plays a role in the metabolism of both adipose tissue (adipocytes) (Kim and Spiegelman, 1996) and skeletal muscle (myocytes) (Guillet-Deniau et al., 2002), it is tempting to speculate that BRG1 could be involved in the regulation of metabolic homeostasis in these tissues as well by virtue of contributing to SREBP maturation. Third, it is noteworthy that despite the decreased presence of nuclear SREBP in the LKO hepatocytes, overall SREBP levels remained undisturbed. A likely explanation could be that the SREBP expression, at the transcriptional level, might be subjected to regulation by BRG1. It has long been documented that both SREBP1 (Amemiya-Kudo et al., 2000) and SREBP2 (Sato et al., 1996) can bind to their respective promoters and activate the transcription *in cis*. Because BRG1 can interact with both SREBP1 (Li et al., 2018) and SREBP2 (Fan et al., 2020), it is possible that reduced FL-SREBP processing in the LKO cells may be offset by a proportional reduction in its expression such that overall FL-SREBP levels remain marginally affected.

In summary, we present evidence to demonstrate that BRG1 can contribute to cellular lipid metabolism by, in addition to acting as an SREBP co-factor, promoting SCAP-dependent SREBP maturation. These data not only place BRG1 in the center of metabolic programming but provide renewed rationale for targeting BRG1 in the intervention of human diseases related to lipid metabolic disorders.

## Data Availability Statement

The original contributions presented in the study are included in the article/Supplementary Material, further inquiries can be directed to the corresponding author/s.

## Ethics Statement

The animal study was reviewed and approved by the Nanjing Medical University Ethics Committee on Humane Treatment of Experimental Animals.

## Author Contributions

ZF and YX conceived the project. MK, YZ, and JS designed the experiments, performed experiments, collected data, and analyzed data. YX wrote the manuscript. ZF handled the funding. All authors contributed to the article and approved the submitted version.

## Conflict of Interest

The authors declare that the research was conducted in the absence of any commercial or financial relationships that could be construed as a potential conflict of interest.
