# Supplementary material for: The Chromatin Remodeling Protein BRG1 Regulates SREBP Maturation by Activating SCAP Transcription in Hepatocytes
Source: Front Cell Dev Biol. 2021 Feb 25;9:622866. doi: 10.3389/fcell.2021.622866 (PMC7947303; doi:10.3389/fcell.2021.622866)
Supplement: Supplementary file 1 [file Data_Sheet_1.PDF]

Kong M *et al*: The chromatin remodeling protein BRG1 regulates SREBP maturation by activating SCAP transcription in hepatocytes

Online supplementary material

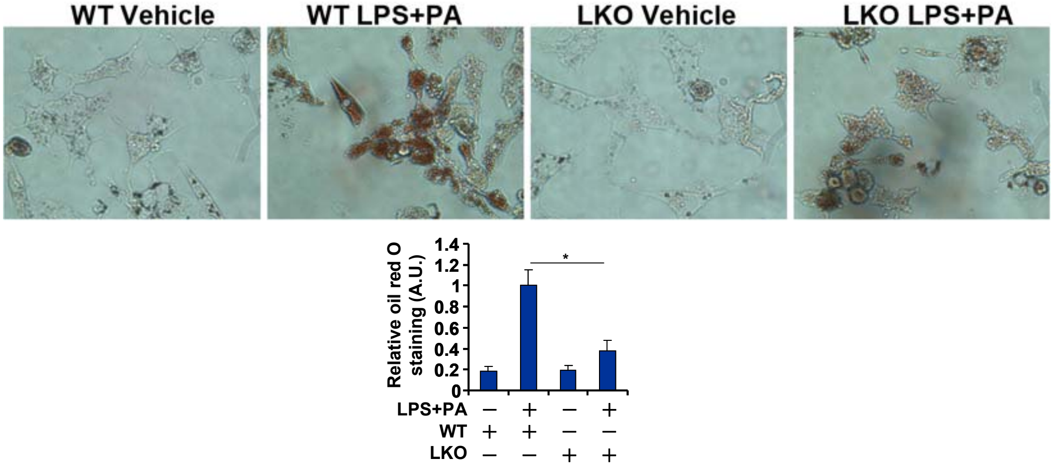

Fig.S1: Primary hepatocytes isolated from WT mice or LKO mice were treated with or without LPS+PA. Lipid droplet was examined by oil red O staining.

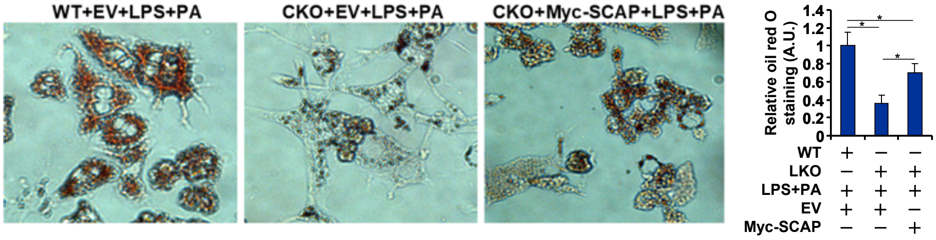

Fig.S2: A Myc-tagged SCAP expression construct was transfected into primary hepatocytes isolated from BRG1 LKO mice followed by treatment with LPS+PA. Lipid droplet was examined by oil red O staining.
